# Impact of Centralisation of Radical Prostatectomy Driven by the Introduction of Robotic Systems on Positive Surgical Margin and Biochemical Recurrence in pT2 Prostate Cancer

**DOI:** 10.1002/cam4.70514

**Published:** 2025-01-17

**Authors:** Ibrahim Ibrahim, Omar Kouli, Sanjana Ilangovan, Melanie Sneddon, Sarika Nalagatla, Carol Marshall, Lorenzo Dutto, Hing Y. Leung, Imran Ahmad

**Affiliations:** ^1^ Department of Urology Queen Elizabeth University Hospital Glasgow UK; ^2^ The Walton Centre Aintree University Hospital Liverpool UK; ^3^ School of Cancer Sciences University of Glasgow Glasgow UK; ^4^ CRUK Scotland Institute Glasgow UK

**Keywords:** biochemical recurrence, positive surgical margin, prostate cancer, radical prostatectomy, robotic surgery

## Abstract

**Background:**

To assess how centralisation of cancer services via robotic surgery influenced positive surgical margin (PSM) occurrence and its associated risk of biochemical recurrence (BCR) in cases of pT2 prostate cancer (PC).

**Methods:**

Retrospective analysis of all radical prostatectomy (RP) cases performed in the West of Scotland during the period from January 2013 to June 2022. Primary outcomes were PSM and BCR. The secondary outcomes compared the impact of centralisation and surgical approach on PSM and BCR; and margin length and location on BCR. Propensity score matching and Cox regression models were performed using R.

**Results:**

A total of, 907 patients were included; 662 robot assisted radical prostatectomy (RARP), 245 open RP. PSM rate was 17.7% (161/907), similar in RARP and open cohorts. Patients with PSM had higher rates of BCR; 26.7%, compared to 8.7% in patients with no PSM. Patients with margins of ≥ 1 mm had higher risk of developing BCR. Patients who underwent open RP had increased incidence of PSM ≥ 1 mm; 40/43 (93%) compared to 83/117 (71%) in robotic approach (*p* = 0.003). Limitations include the study being retrospective, introduction of centralisation and robot concurrently, and evolution of practice.

**Discussion:**

PSMs in pT2 PC are associated with higher rates of BCR. Introduction of centralisation via the robot had no impact on PSM occurrence or BCR, although did demonstrate a reduction in PSM length.

## 1 Introduction

Prostate cancer (PC) is the most common malignancy among males in the United Kingdom, with an escalating trend in early‐stage diagnoses [1]. Management strategies for localised PC have evolved, encompassing active surveillance, radical surgery, focal therapies and radiotherapy [2]. The landscape of service delivery in the United Kingdom and Europe has undergone significant transformations, marked by the introduction of robotic surgery as the ‘standard of care’ for radical prostatectomy and the de facto centralisation of cancer treatment due to the cost of these systems [3].

The introduction of the Da Vinci Xi robotic system to the West of Scotland in 2016, resulted in the centralisation of radical prostatectomy (RP) surgery to a single centre, aimed to revolutionise cancer care for PC in a population exceeding 3 million [4]. Systematic reviews have indicated improved perioperative outcomes and a potential reduction in positive margins with the adoption of robotic systems [5]. It has been believed to be economically beneficial in high volume centres, with over 150 cases per year [6].

Almost a decade later, as robotic systems become increasingly accessible across the National Health Service (NHS) to smaller units, discussions about decentralising services to units equipped with robotic systems and adequate expertise are underway. Current data suggests a rising trend in RP, even for higher‐risk PC cases [7].

Positive surgical margin (PSM) on prostate specimens post‐RP is acknowledged as a predictor for biochemical recurrence (BCR), defined as a detectable PSA reading of ≥ 0.1 ng/mL on two consecutive tests [8]. PSM rates are influenced by various factors, including T stage and Gleason score/International Society of Urological Pathology (ISUP) grade [9]. Patients with PSM face an elevated risk of BCR, disease progression, requiring additional treatment with the associated costs and morbidity. This emphasises the importance of identifying prognostic predictors for post‐RP outcomes in discussions with PC patients considering surgery [10, 11].

While several studies have established a positive correlation between PSM and BCR, others have demonstrated limited significance [10, 12, 13]. Varied PSM occurrence rates, ranging from 11% to 38%, have been reported in the literature, with higher T stage increasing the risk of PSMs [10]. Beckmann et al. found that PSM rates were 18%, 35% and 54% in T2, T3a and T3b PC respectively [14]. Shikanov et al. also reported 11% and 41% rates of PSM in T2 and T3 PC respectively, with a correlation between PSM length and BCR rates [15].

In our study we examined how centralisation of PC surgery via robotic systems affected oncological outcomes in localised disease. We hypothesised that centralisation would reduce the rates of pathological T stage 2 (pT2) PSM, the length of the PSM and subsequent BCR rates.

## 2 Methods

In this prospective dataset we were able to access patients who underwent RP between January 2013 and June 2022 in the West of Scotland across five NHS health boards. All patients who had pT2 staging were included. This analysis was performed according to STROBE reporting guidelines for observational studies [16].

The primary outcome was PSM and subsequent BCR. The secondary outcomes compared the impact of centralisation and surgical approach on PSM and BCR; and margin length and location on BCR. Data on age, pre‐operative prostate specific antigen (PSA) levels and surgical approach. From our centralised pathological database, we obtained margin status, presence/absence of perineural invasion and Gleason score/ISUP grade. Margin status was divided into apical, basal and circumferential margin involvement, with the length of PSM also recorded.

### 2.1 Statistical Analysis

Continuous data are presented as medians (IQRs). Categorical data is presented as percentage frequencies. For univariate analysis, Mann–Whitney *U*‐test was used for comparison of continuous data and Fisher's exact test for categorical data.

Multivariable Cox proportional hazards regression was performed to determine whether the presence of PSM was associated independently with the occurrence of BCR. Proportional hazards assumption was tested with the Schoenfeld residuals. Multivariable logistic regression was performed to determine whether the use of robotic approach was independently associated with positive surgical margins.

To investigate the association between PSM and BCR, propensity score matching was used to minimise selection bias in terms of who did or did not have a PSM. The propensity score was defined as the probability that a patient would have a PSM. Unlike nearest‐neighbour propensity score matching approaches, which can lead to inappropriate discarding of patient data, full matching was used to allow multiple patients from each group to be matched together (if appropriate) and weighted to achieve balance. The balance in clinical factors between groups was assessed before and after using the absolute standardised mean difference, and a value below 0.2 was considered to indicate that a variable was well‐balanced between groups [17, 18]. Subsequent doubly robust estimation was performed through risk adjustment using multivariable Cox regression model, based on the same variables as used to generate the propensity score.

Sensitivity analyses were also performed to investigate the association between PSM length (< 1 mm and ≥ 1 mm) and location (apical, basal and circumferential) on BCR.

All effect estimates are presented as odds ratios (ORs) for binary outcome data and hazard ratios (HRs) for time‐to‐event data, with 95% confidence intervals. The threshold for statistical significance was set a priori as *p* < 0.050. All analyses were undertaken using R version 4.1 (R Foundation for Statistical Computing, Vienna, Austria).

## 3 Results

### 3.1 Observation of Cohorts

Of the 1708 patients who underwent RP during the study period, 907 (53%) were found to have pT2 disease. The mean time for follow‐up was 3.85 years (range 0.19–10.7 years) with a mean time to BCR of 2.48 years (range 34 days–10.1 years). The median age was 64 (range 44–79), the median PSA was 7 (range 1–54). Prior to centralisation in January 2016, there were six hospitals performing RP with seven surgeons. The average consultant volume per year was 20 (median of 10.5 cases per year, range 2–42). With centralisation, RARP has been performed by three surgeons in a single centre with an average consultant volume of 58 per year (median 53.5 cases per year, range 17–115).

The absolute standardised mean deviation (aSMD) was analysed and was less than 0.2 for each variable indicated a well‐balanced comparison. Table 1 demonstrates the matched characteristics on PSM.

**TABLE 1:** Balance table for characteristics of patients before and after propensity score matching on positive surgical margin.

	Unmatched characteristics			Propensity score matched characteristics		
	No	Yes	aSMD^a^	No	Yes	aSMD^a^
Age						
Mean (SD)	63.4 (6.34)	63.7 (6.47)	0.012	63.7 (6.24)	63.7 (6.47)	0.001
PSA ng/mL						
Mean (SD)	9.19 (5.81)	9.54 (6.15)	0.012	9.41 (6.13)	9.54 (6.15)	0.001
PNI						
No	191 (26.0)	30 (18.9)	0.17	135 (18.3)	30 (18.9)	0.014
Yes	545 (74.0)	129 (81.1)		601 (81.7)	129 (81.1)	
ISUP (Post‐operative)						
1	128 (17.4)	25 (15.7)	0.099	145 (19.7)	25 (15.7)	0.111
2	457 (62.1)	104 (65.4)		452 (61.5)	104 (65.4)	
3	118 (16.0)	24 (15.1)		107 (14.6)	24 (15.1)	
4	22 (3.0)	3 (1.9)		15 (2.0)	3 (1.9)	
5	11 (1.5)	3 (1.9)		16 (2.2)	3 (1.9)	
Approach						
Open RP	200 (27.2)	43 (27.0)	0.003	221 (30.0)	43 (27.0)	0.066
RARP	536 (72.8)	116 (73.0)		515 (70.0)	116 (73.0)	

^a^ Absolute standardised mean difference.

The total dataset was 907, the number of patients included in the model was 895. This was because 12 (1.3%) of patients had missing PSA levels. We have checked for associations between missing and observed data for PSA (see table below) and found that the data was missing completely at random (MCAR). Since the missingness was MCAR and missing data was low (1.3%), we have done a complete case analysis for the regression model. This is an accepted method for dealing with MCAR data known in literature [19].

### 3.2 Effect of Variables on BCR

Overall, 17.7% (*n* = 161/907) of patients had a PSM and 108 (12%) exhibited BCR (Table 2). Patients with PSM had significantly higher rates of BCR compared to those who did not have PSM (26.7% (*n* = 43/161) and 8.7% (*n* = 65/746), *p* < 0.001, respectively). Three quarters of the patients had perineural invasion (PNI) (75.5%, *n* = 685/907) and the most predominant ISUP grade was 2 (62.4%, *n* = 566/907). Patients with higher ISUP grades also had higher BCR rates compared to lower ISUP grades. There were no statistically significant differences in BCR rates in relation to age, PSA, robotic approach and perineural invasion (Table 2).

**TABLE 2:** Patient, surgical and pathological cohort characteristics by biochemical recurrence.

	Biochemical recurrence			
	No	Yes	Total	*p*
Total *N* (%)	799 (88.1)	108 (11.9)	907	
Age (years)				
Median (IQR)	64.0 (59.0–68.0)	65.0 (60.0–69.0)	64.0 (59.0–68.0)	0.290
Prostate specific antigen ng/mL (PSA)				
Median (IQR)	7.5 (5.5–11.3)	8.1 (5.6–11.1)	7.5 (5.6–11.3)	0.628
Robotic approach				
No	212 (86.5)	33 (13.5)	245	0.419
Yes	587 (88.7)	75 (11.3)	662	
Positive surgical margin				
No PSM	681 (91.3)	65 (8.7)	746	< 0.001
PSM	118 (73.3)	43 (26.7)	161	
Positive surgical margin length (mm)				
No PSM	681 (91.2)	66 (8.8)	746	< 0.001
PSM < 1 mm	33 (89.2)	4 (10.8)	37	
PSM ≥ 1 mm	85 (69.1)	38 (30.9)	123	
Apical positive surgical margin				
No	739 (89.7)	85 (10.3)	824	< 0.001
Yes	60 (72.3)	23 (27.7)	83	
Basal positive surgical margin				
No	779 (88.5)	101 (11.5)	880	0.033
Yes	20 (74.1)	7 (25.9)	27	
Circumferential positive surgical margin				
No	747 (90.0)	83 (10.0)	830	< 0.001
Yes	52 (67.5)	25 (32.5)	77	
Perineural invasion				
No	195 (87.8)	27 (12.2)	222	0.905
Yes	604 (88.2)	81 (11.8)	685	
ISUP (post‐operative)				
1	142 (92.2)	12 (7.8)	154	< 0.001
2	518 (91.5)	48 (8.5)	566	
3	119 (81.0)	28 (19.0)	147	
4	17 (65.4)	9 (34.6)	26	
5	3 (21.4)	11 (78.6)	14	

**FIGURE 1:**
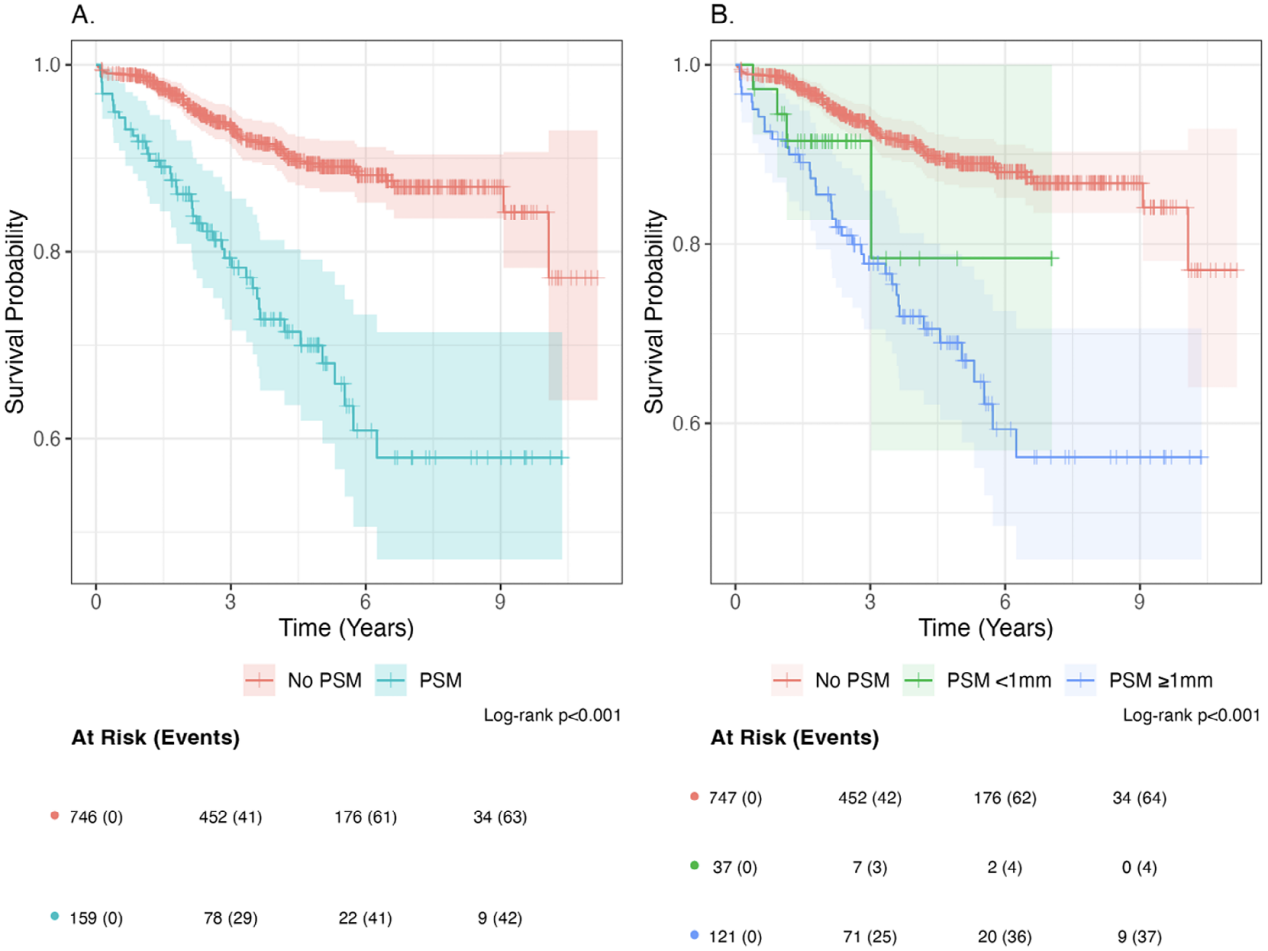
Kaplan–Meier curves for biochemical recurrence (BCR) stratified by (A) presence or absence of positive surgical margin (PSM) and (B) PSM length.

Propensity score matching produced balanced, well matched treatment groups balancing characteristics including age, PSA, PNI and ISUP optimising validity with the analysis. On univariable analysis, patients with a PSM had significantly higher rates of BCR compared to those with no PSM (HR 3.46, 95% CI 2.33–5.12; *p* < 0.001) (Figure 1A and Table 2). This persisted on Cox proportional hazard regression which demonstrated a higher hazard of BCR for patients with PSM (HR 3.99, 2.67–5.98; *p* < 0.001). This association was maintained following propensity score matching as patients with PSM were more than three times likely to develop BCR than those without (HR 3.39, 2.18–5.26; *p* < 0.001) (Table 3). Univariable and multivariable HR represents unmatched estimates demonstrated in Table 3.

**TABLE 3:** Factors associated with the rate of biochemical recurrence following propensity score matching on positive surgical margin presence and risk adjustment.

	Biochemical recurrence				
	No	Yes	Unmatched univariable HR (95% CI)	Unmatched multivariable HR (95% CI)	Matched HR (95% CI)
Positive surgical margin					
No	672 (85.2)	64 (60.4)	1.00 (reference)	1.00 (reference)	1.00 (reference)
Yes	117 (14.8)	42 (39.6)	3.46 (2.33–5.12, *p* < 0.001)	3.99 (2.67–5.98, *p* < 0.001)	3.39 (2.18–5.26, *p* < 0.001)
Age (years)					
Mean (SD)	63.4 (6.4)	63.9 (6.3)	1.02 (0.99–1.05, *p* = 0.264)	0.99 (0.96–1.03, *p* = 0.645)	0.96 (0.93–1.00, *p* = 0.078)
Prostate specific antigen ng/mL (PSA)					
Mean (SD)	9.3 (5.9)	9.2 (5.3)	1.00 (0.97–1.03, *p* = 0.995)	0.99 (0.95–1.03, *p* = 0.536)	0.99 (0.95–1.04, *p* = 0.770)
Perineural invasion					
No	194 (24.6)	27 (25.5)	1.00 (reference)	1.00 (reference)	1.00 (reference)
Yes	595 (75.4)	79 (74.5)	1.20 (0.77–1.87, *p* = 0.412)	0.91 (0.57–1.44, *p* = 0.679)	1.15 (0.54–2.41, *p* = 0.720)
ISUP (post‐operative)					
1	141 (17.9)	12 (11.3)	1.00 (reference)	1.00 (reference)	1.00 (reference)
2	514 (65.1)	47 (44.3)	1.25 (0.66–2.36, *p* = 0.497)	1.32 (0.69–2.52, *p* = 0.402)	1.14 (0.59–2.22, *p* = 0.700)
3	115 (14.6)	27 (25.5)	2.96 (1.50–5.86, *p* = 0.002)	3.43 (1.69–6.94, *p* = 0.001)	2.39 (0.98–5.83, *p* = 0.057)
4	16 (2.0)	9 (8.5)	6.02 (2.53–14.30, *p* < 0.001)	7.90 (3.27–19.05, *p* < 0.001)	21.59 (6.19–75.34, *p* < 0.001)
5	3 (0.4)	11 (10.4)	18.95 (8.28–43.37, *p* < 0.001)	22.99 (9.59–55.11, *p* < 0.001)	32.73 (10.03–106.84, *p* < 0.001)

### 3.3 Margin Involvement on BCR

Of the PSM patients, 83 (52%) had apical involvement, 27 (17%) had basal involvement and 77 (48%) had circumferential involvement. 23 (28%) patients of those with apical involvement demonstrated BCR, 7 (26%) of those with basal involvement demonstrated BCR and 25 (32%) of those with circumferential involvement demonstrated BCR. On sensitivity analysis looking at the location of the PSM on BCR, both apical and circumferential margins yielded hazard risks of over three folds on Cox proportional hazard regression (HR 3.45, 2.15–5.52; *p* < 0.001 and HR 3.79, 2.39–6.01; *p* < 0.001). However, basal PSM did not have a statistically significant association with BCR compared to patients with no PSM (HR 1.81, 0.72–4.58; *p* = 0.208) (Table S1).

### 3.4 Margin Length on BCR

Of the 160 PSM patients who had a length reported, 123 (77%) had a PSM length of 1 mm or more and 37 (23%) had a PSM less than 1 mm. 38/123 (31%) of patients who had a PSM length of 1 mm or more experienced BCR compared to 4/37 (11%) of those with a PSM length less than 1 mm. Comparing PSM lengths on BCR, patients with a margin of < 1 mm had no statistically significant difference compared to patients with no PSM on univariate analysis (HR 2.16, 0.78–5.95; *p* = 0.138) (Figure 1b and Table S2). On the other hand, patients with margins of ≥ 1 mm had a significantly higher risk of developing BCR compared to patients with no PSM on univariate analysis (HR 3.59, 2.40–5.38; *p* < 0.001) (Figure 1b and Table S2). However, on Cox proportional hazard regression, both groups with PSMs (< 1 mm and ≥ 1 mm) demonstrated statistically higher hazards of BCR (HR 3.09, 1.11–8.63; *p* = 0.031 and HR 3.93, 2.59–5.97; *p* < 0.001, respectively).

#### 3.4.1 Comparing Open RP and RARP, on PSM and BCR Outcomes

Over two thirds of patients with pT2 PC (72.9%, *n* = 662/907) underwent a RARP (Table 4). Patients undergoing RARP had similar rates of PSMs compared to those who did not undergo surgery via a robotic approach (17.8% (*n* = 118/662) and 17.6% (*n* = 43/245), respectively). This persisted on multivariate logistic regression (OR 0.91, 0.61–1.37; *p* = 0.646). There was no significant difference in BCR when comparing the RARP group, with 75/662 (11.3%) exhibiting BCR, to the open RP cohort, with 33/245 (13.5%) exhibiting BCR (*p* = 0.357).

**TABLE 4:** Univariate and multivariate logistic regression models of the association between robotic approach and positive surgical margin.

	No PSM	PSM	OR (univariable)	OR (multivariable)
Robotic approach				
No	202 (82.4)	43 (17.6)	1.00 (reference)	1.00 (reference)
Yes	544 (82.2)	118 (17.8)	1.02 (0.70–1.51, *p* = 0.924)	0.91 (0.61–1.37, *p* = 0.646)
Age (years)				
Mean (SD)	63.4 (6.3)	63.7 (6.4)	1.01 (0.98–1.04, *p* = 0.564)	1.01 (0.98–1.04, *p* = 0.661)
Prostate specific antigen ng/mL (PSA)				
Mean (SD)	9.2 (5.8)	9.5 (6.2)	1.01 (0.98–1.04, *p* = 0.494)	1.01 (0.98–1.04, *p* = 0.586)
Perineural invasion				
No	192 (86.5)	30 (13.5)	1.00 (reference)	1.00 (reference)
Yes	554 (80.9)	131 (19.1)	1.51 (1.00–2.36, *p* = 0.059)	1.53 (0.98–2.44, *p* = 0.069)
ISUP (post‐operative)				
1	129 (83.8)	25 (16.2)	1.00 (reference)	1.00 (reference)
2	460 (81.3)	106 (18.7)	1.19 (0.75–1.95, *p* = 0.477)	1.02 (0.62–1.71, *p* = 0.940)
3	123 (83.7)	24 (16.3)	1.01 (0.54–1.86, *p* = 0.983)	0.90 (0.47–1.71, *p* = 0.745)
4	23 (88.5)	3 (11.5)	0.67 (0.15–2.13, *p* = 0.543)	0.61 (0.14–1.96, *p* = 0.450)
5	11 (78.6)	3 (21.4)	1.41 (0.30–4.90, *p* = 0.619)	1.13 (0.24–4.03, *p* = 0.858)

Within the open RP cohort, apical involvement was seen in 23/43 (53.5%), basal involvement in 8/43 (18.6%) and circumferential involvement in 19/43 (44.2%) of specimens. Similarly, in the RARP cohort, 60/118 (50.8%) had apical involvement, 19/118 (16.1%) had basal involvement and 58/118 (49.2%) had circumferential involvement. The univariate and multivariate logistic regression models examining the association between the robotic approach and positive surgical margins (PSM) are presented in Table 4.

On sensitivity analysis comparing the use of robotic approach on the different PSM groups (< 1 mm and ≥ 1 mm), it was found that the use of the robot had lower rates of PSMs ≥ 1 mm, 83/117 (71%), compared to open RP cohort, 40/43 (93%), (adjusted OR 0.15, 0.030.46; *p* = 0.003) (Table S3). Thirteen (5%) had BCR in the open RP cohort PSM patients compared to 30 (5%) who had RARP (*p* = 0.426). The corresponding BCR rates were 31%, 9%, 30% and 33% for RARP and PSM greater than 1 mm, RARP and PSM less than 1 mm, open RP approach and PSM greater than 1 mm and open RP approach and PSM less than 1 mm (*p* = 0.733).

## 4 Discussion

We present data derived from a decade‐long study encompassing the transition from conventional practices to the incorporation of a robotic system in the management of pT2 PC patients in the West of Scotland. PSM were identified in 18% of pT2 PC specimens, a prevalence consistent across both the robotic and non‐robotic cohorts (18%).

Since 2012, all cancer care in Scotland has been subject to quality performance indicators (QPIs). The overarching aim of this cancer quality work programme is to ensure that activity at NHS board level is focused on areas most important in terms of improving survival and patient experience whilst reducing variance and ensuring safe, effective and personalised cancer care [18, 20].

Our findings demonstrate a substantial association between PSM and an elevated risk of BCR in pT2 PC, with a BCR rate of 27% observed in cases with PSM compared to 9% in those without (*p* < 0.001). Further analysis of PSM lengths revealed a significantly heightened risk of BCR when the PSM was 1 mm or greater. Of the examined patients with PSM, 31% with a PSM of 1 mm or greater experienced BCR, contrasting with 11% among those with a PSM length less than 1 mm. These insights contribute valuable considerations for patient discussions regarding PSMs in pT2 PC at post‐operative consultations.

Interestingly, the introduction of robotic‐assisted procedures with the centralisation of services exhibited no discernible impact on PSM occurrence in pT2 disease, maintaining an overall PSM rate of 18% in both cohorts. Notably, open RP approach demonstrated a higher incidence (93%) of PSM greater than or equal to 1 mm compared to the RARP group (71%). Although no immediate correlation with BCR was observed in our analysis, this warrants further exploration with a more extensive patient subgroup, considering the acknowledged association between BCR and PSM.

A significant health system restructuring in 2016 centralised RP services within the West of Scotland Cancer Network (WoSCAN), resulting in a median consultant volume of 53.5 RPs per year, a notable increase from the previous median of 10.5 per year. This organisational and technological restructure, including the utilisation of the Da Vinci robotic system, demonstrated improvements solely in PSM length within our patient cohort.

Our data highlights the apical margin as the most involved margin (seen in 52% of specimens), irrespective of robotic assistance. Previous studies also demonstrated that the apical margin is the most involved [21, 22]. Our analysis also demonstrated that an apical or circumferential PSM, is associated with a significantly higher risk of BCR (*p* < 0.001). Similarly, Eastham et al. demonstrated that solely posterior‐lateral PSMs (equivalent of circumferential margins) were previously associated with higher risks of BCR in 2007 [19, 21].

The ISUP grade correlated directly with higher risk of BCR. The rates of BCR were 7.8%, 8.5%, 19.0%, 34.6% and 78.6% for ISUP grades 1, 2, 3, 4 and 5, respectively. Although this has previously been reported in studies, our study presents the largest cohort size of such analysis [23]. Our study concluded that perineural invasion, age and pre‐operative PSA demonstrated no significance in risk of BCR.

A limitation of our study includes that the two variables that may have independently contributed to the outcomes; centralisation of services and introduction of the robotic system were implemented simultaneously. This is representative of many centres across the United Kingdom and Europe, driven by cost of purchasing the robotic system. As various robotic systems are coming to market, and the cost is falling, there is the desire in many smaller urology departments to repatriate cancer surgeries such as RARP, to help better serve local patient populations and boost staff recruitment in these units [24, 25].

Although our study presents an extensive cohort of patients, a limitation is that it was a case series from a retrospective cohort and not a randomised control trial (RCT), which would not be feasible to perform as there are nearly no further open RPs performed. Another limitation includes the changes in oncological management during the study period, including the publication of the RADICALS‐RT study which demonstrated there was no advantage of adjuvant over salvage radiotherapy [26]. This also applies to changes in diagnostic evaluation and management pathways that have developed over the last decade, may confound the results observed.

Our data provides a comprehensive review of surgical and corresponding oncological outcomes in patients with pT2 PC, however data regarding quality of life (QoL) remains inaccurately recorded. This provides a limitation to reliably evaluate the difference between the cohorts with regards to their functional outcomes such as erectile function and urinary incontinence post‐operatively. Previous studies have demonstrated mixed conclusions regarding any difference in rates of erectile and urinary function with utilisation of robotic systems [27, 28, 29, 30]. Missing values in the observed variables were limited to a single PSM that did not have the length reported, this has therefore not been included in the comparison of PSM length on BCR. Additionally, some PSA values were missing in the original database and these were manually extracted from patient records. Twelve patients did not have a pre‐operative PSA level recorded in the patient notes and this was taken into consideration as discussed above.

Consideration of learning curves and experience should also be appreciated as with the introduction of the robot, with the initial patients performed during a learning curve of the three surgeons. This was not the case with the open RP cohort since all were established surgeons in 2013.

In conclusion, our study demonstrates a PSM rate of 17% in pT2 PC. PSM in pT2 PC is associated with higher rates of BCR. A PSM of 1 mm or greater correlates with a higher risk of BCR. Introduction of centralisation via robot surgery had no impact on PSM occurrence or BCR, although did demonstrate a reduction in PSM length during the follow‐up duration of our study. It remains unknown whether a difference exists with longer term follow‐up for BCR.

## Author Contributions

**Ibrahim Ibrahim:** data curation (lead), formal analysis (lead), writing – original draft (equal). **Omar Kouli:** data curation (equal), formal analysis (equal), methodology (equal). **Sanjana Ilangovan:** data curation (equal). **Melanie Sneddon:** data curation (equal). **Sarika Nalagatla:** data curation (equal). **Carol Marshall:** data curation (equal). **Lorenzo Dutto:** project administration (supporting). **Hing Y. Leung:** supervision (supporting). **Imran Ahmad:** conceptualization (lead), data curation (equal), formal analysis (equal), funding acquisition (lead), investigation (equal), methodology (equal), project administration (lead), writing – original draft (equal), writing – review and editing (lead).

## Ethics Statement

This study was approved by the Local NHS Ethical Committee, with prior written informed consent obtained from study participants. The study was performed in accordance with the Declaration of Helsinki.

## Conflicts of Interest

The authors declare no conflicts of interest.

## Supporting information


Table S1.

## Data Availability

The datasets generated during and/or analysed during the current study are not publicly available due confidential patient data but are available from the corresponding author on reasonable request.
