# Supplementary material for: Impact of Centralisation of Radical Prostatectomy Driven by the Introduction of Robotic Systems on Positive Surgical Margin and Biochemical Recurrence in pT2 Prostate Cancer
Source: Cancer Med. 2025 Jan 17;14(2):e70514. doi: 10.1002/cam4.70514 (PMC11756548; doi:10.1002/cam4.70514)
Supplement: Supplementary file 1 — Table S1. [file CAM4-14-e70514-s001.docx]

**Supplementary Table 1: Sensitivity analysis: Summary of univariate and multivariate Cox proportional regression models of the association between positive surgical margin location and biochemical recurrence**

| Margin specified | Biochemical Recurrence | | HR (univariable) | HR (multivariable) |
| --- | --- | --- | --- | --- |
|  | No (%) | Yes (%) |  |  |
| Apical positive surgical margin | 824 (90.8) | 83 (9.2) | 1.00 (reference) | 1.00 (reference) |
|  |  |  | 3.16 (1.99-5.02, p<0.001) | 3.45 (2.15-5.52, p<0.001) |
| Basal positive surgical margin | 880 (97.0) | 27 (3.0) | 1.00 (reference) | 1.00 (reference) |
|  |  |  | 1.98 (0.86-4.52, p=0.106) | 1.81 (0.72-4.58, p=0.208) |
| Circumferential positive surgical margin | 830 (91.5) | 77 (8.5) | 1.00 (reference) | 1.00 (reference) |
|  |  |  | 3.90 (2.49-6.11, p<0.001) | 3.79 (2.39-6.01, p<0.001) |

**Supplementary Table 2: Sensitivity analysis: Univariate and multivariate Cox proportional regression models of the association between positive surgical margin length and biochemical recurrence**

| Dependent: Biochemical recurrence |  | Biochemical Recurrence BCR | | | Total N (%) | HR (univariable) | HR (multivariable) |
| --- | --- | --- | --- | --- | --- | --- | --- |
|  |  | No | | Yes |  |  |  |
| Positive surgical margin length (mm) | No PSM | 681 (91.2) | 65 (8.8) | | 746 (82.3) | 1.00 (reference) | 1.00 (reference) |
|  | PSM <1mm | 33 (89.2) | 4 (10.8) | | 37 (4.1) | 2.21 (0.78-5.95, p=0.127) | 3.18 (1.11-8.63, p=0.027) |
|  | PSM ≥1mm | 85 (69.1) | 38 (30.9) | | 123 (13.6) | 3.59 (2.40-5.38, p<0.001) | 3.93 (2.59-5.97, p<0.001) |
| Age (years) | Median (IQR) | 64.0 (59.0 to 68.0) | 65.0 (60.0 to 69.0) | | 63.4 (6.4) | 1.02 (0.99-1.05, p=0.229) | 0.99 (0.96-1.03, p=0.646) |
| Prostate Specific Antigen ng/ml (PSA) | Median (IQR) | 7.5 (5.5 to 11.3) | 8.1 (5.6 to 11.1) | | 9.3 (5.9) | 1.00 (0.97-1.03, p=0.995) | 0.99 (0.95-1.03, p=0.548) |
| Perineural invasion | No | 195 (87.8) | 27 (12.2) | | 222 (24.5) | 1.00 (reference) | 1.00 (reference) |
|  | Yes | 604 (88.2) | 81 (11.8) | | 685 (75.5) | 1.21 (0.78-1.88, p=0.390) | 0.91 (0.57-1.44, p=0.686) |
| ISUP (Post-operative) | 1 | 142 (92.2) | 12 (7.8) | | 154 (17.0) | 1.00 (reference) | 1.00 (reference) |
|  | 2 | 518 (91.5) | 48 (8.5) | | 566 (62.4) | 1.28 (0.68-2.41, p=0.454) | 1.32 (0.69-2.53, p=0.397) |
|  | 3 | 119 (81.0) | 28 (19.0) | | 147 (16.2) | 2.95 (1.50-5.81, p=0.002) | 3.41 (1.68-6.89, p=0.001) |
|  | 4 | 17 (65.4) | 9 (34.6) | | 26 (2.9) | 5.80 (2.44-13.80, p<0.001) | 7.77 (3.22-18.74, p<0.001) |
|  | 5 | 3 (21.4) | 11 (78.6) | | 14 (1.5) | 18.97 (8.29-43.40, p<0.001) | 22.55 (9.41-54.06, p<0.001) |

**Supplementary Table 3: Sensitivity analysis: Univariate and multivariate logistic regression models of the association between robotic approach and positive surgical margin length**

|  |  | PSM <1mm | PSM ≥1mm | OR (univariable) | OR (multivariable) |
| --- | --- | --- | --- | --- | --- |
| Robotic approach | No | 3 (7.0) | 40 (93.0) | - | - |
|  | Yes | 34 (29.1) | 83 (70.9) | 0.18 (0.04-0.55, p=0.007) | 0.15 (0.03-0.46, p=0.003) |
| Age (years) | Mean (SD) | 63.3 (6.6) | 63.9 (6.4) | 1.01 (0.96-1.07, p=0.619) | 1.00 (0.94-1.06, p=0.902) |
| Prostate Specific Antigen ng/ml (PSA) | Mean (SD) | 9.2 (4.5) | 9.7 (6.6) | 1.01 (0.96-1.09, p=0.669) | 1.02 (0.96-1.10, p=0.559) |
| Perineural invasion | No | 8 (26.7) | 22 (73.3) | - | - |
|  | Yes | 29 (22.3) | 101 (77.7) | 1.27 (0.49-3.05, p=0.610) | 1.70 (0.60-4.66, p=0.304) |
| ISUP (Post-operative) | <3 | 33 (25.4) | 97 (74.6) | - | - |
|  | ≥3 | 4 (13.3) | 26 (86.7) | 2.21 (0.79-7.90, p=0.167) | 2.21 (0.73-8.35, p=0.193) |
